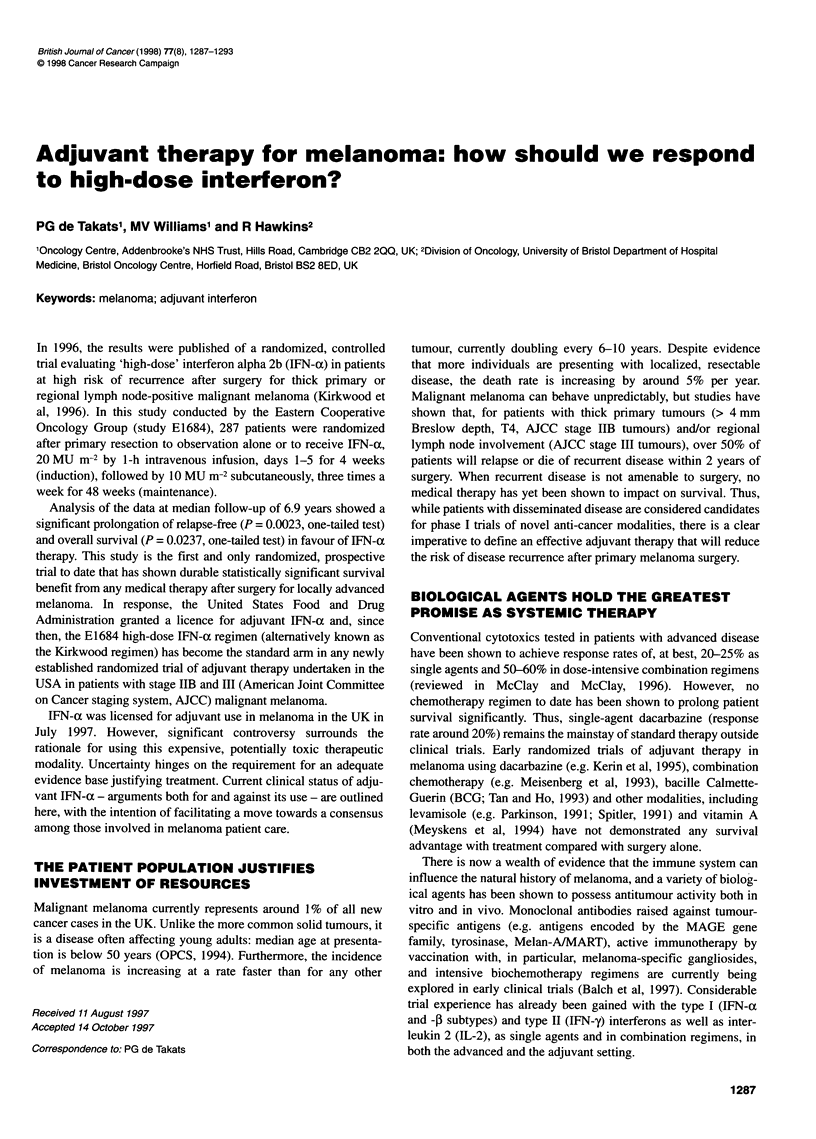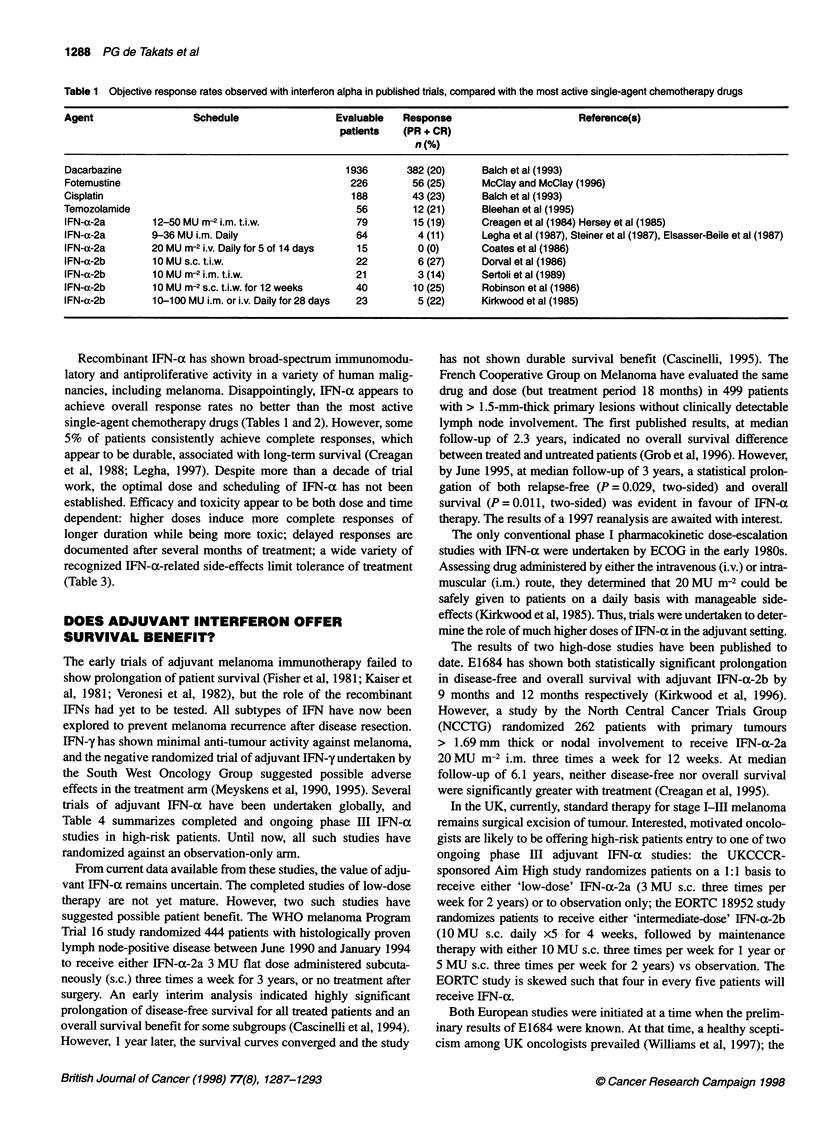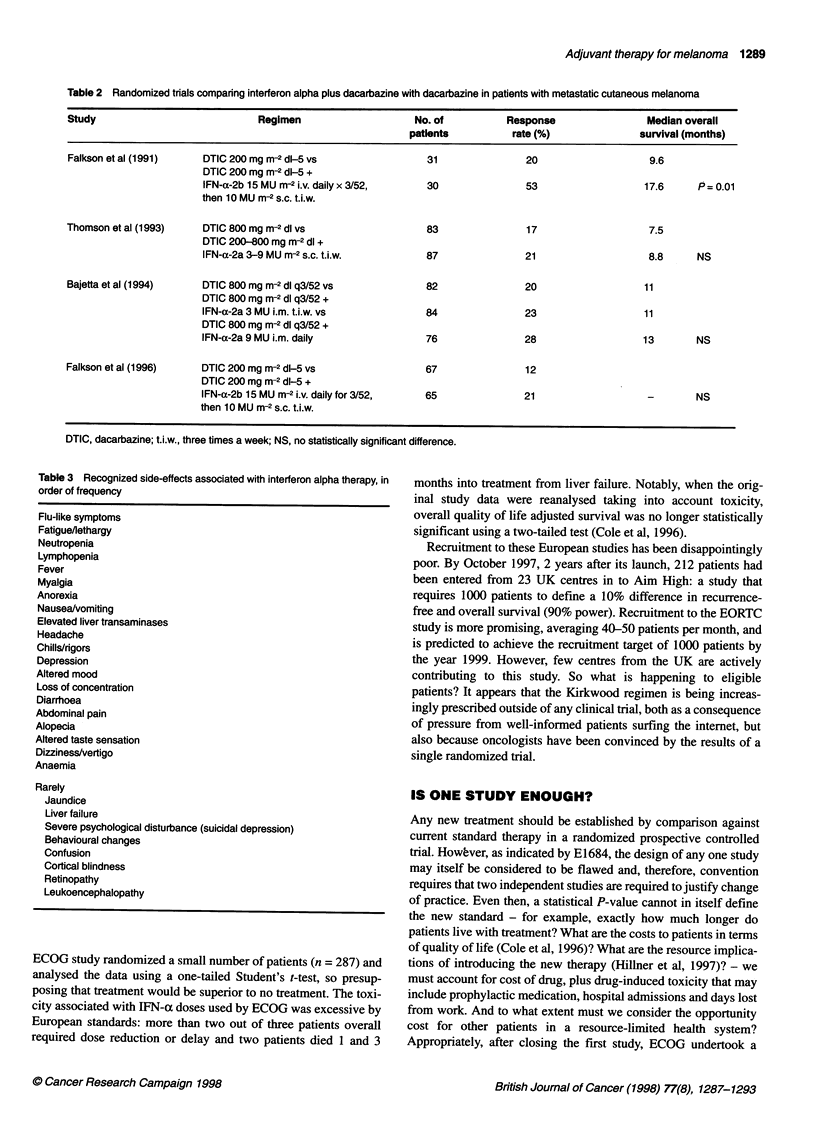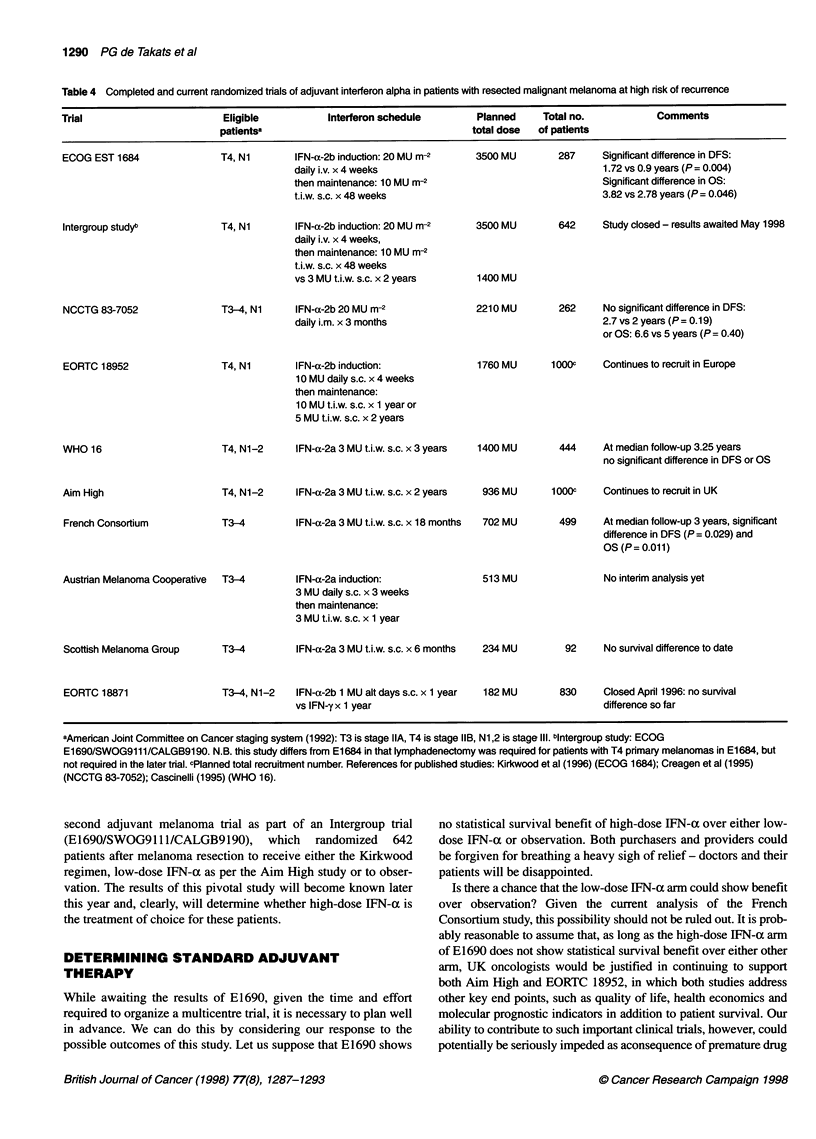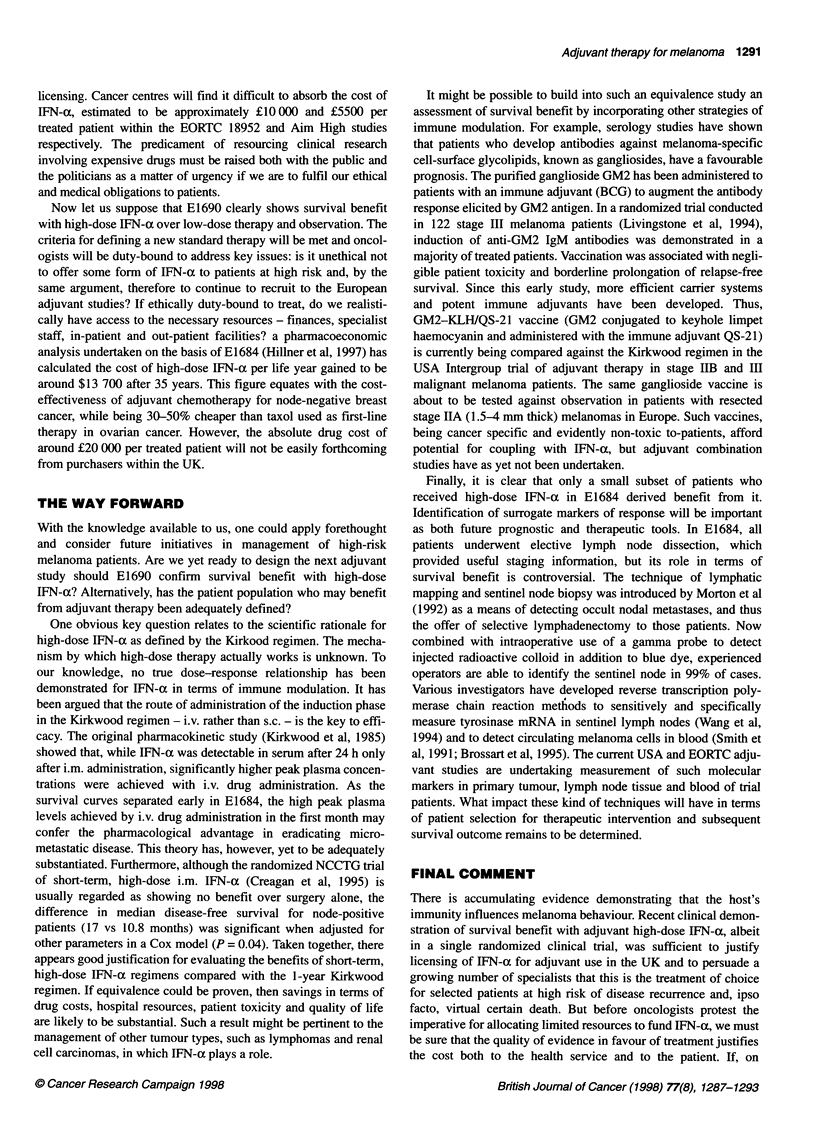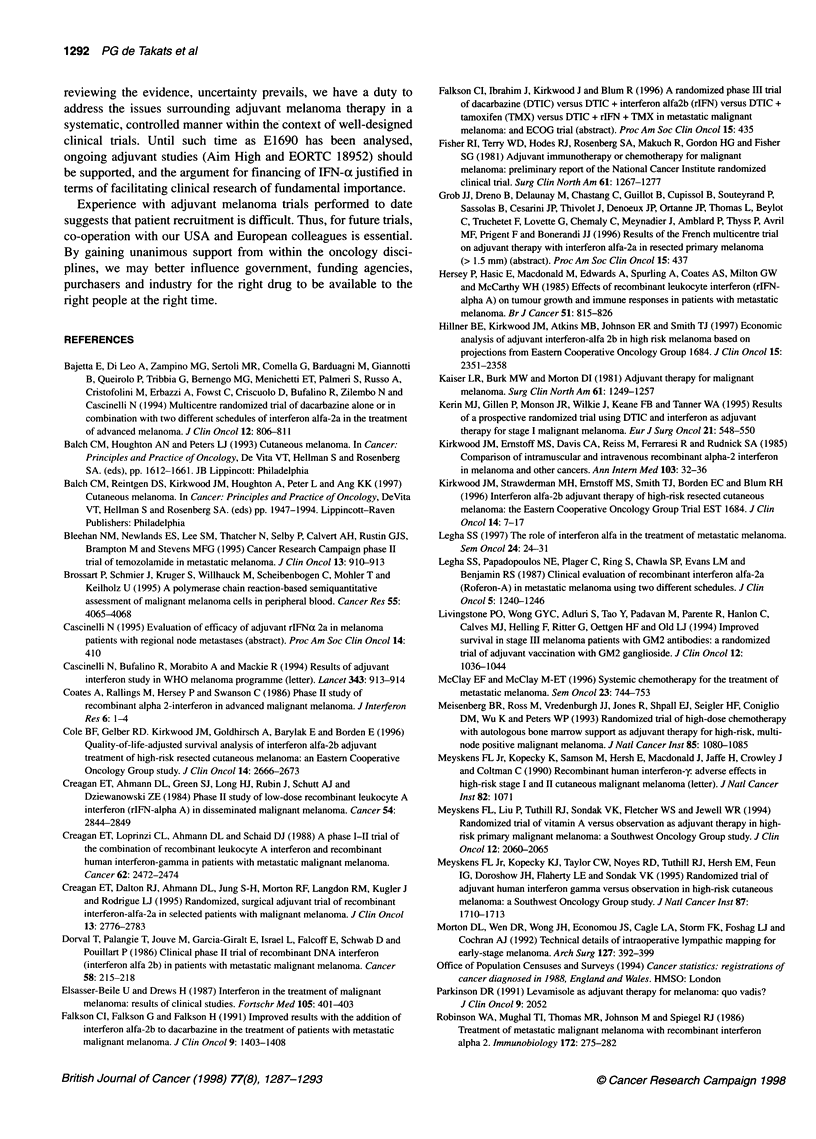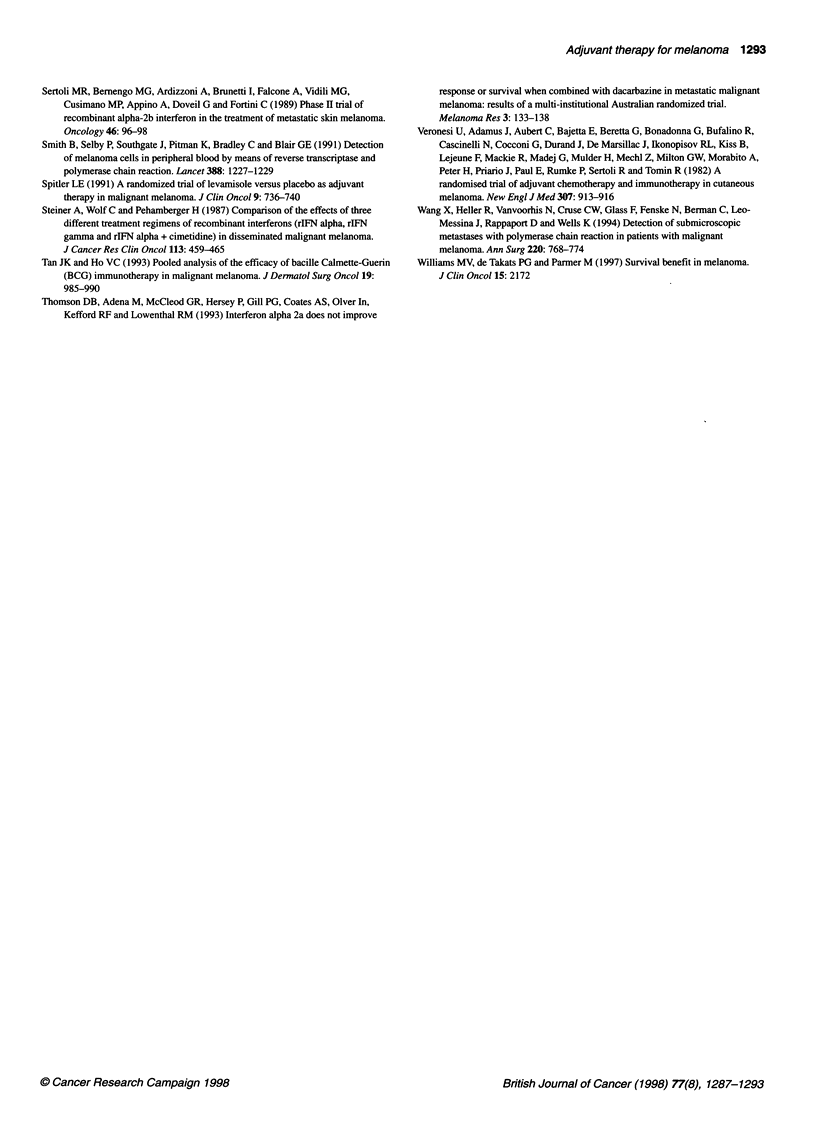# Adjuvant therapy for melanoma: How should we respond to high-dose interferon?

**DOI:** 10.1038/bjc.1998.215

**Published:** 1998-04

**Authors:** P. G. de Takats, M. V. Williams, R. Hawkins

**Affiliations:** Oncology Centre, Addenbrooke's NHS Trust, Cambridge, UK.


British Journal of Cancer (1998) 77(8), 1287–1293
© 1998 Cancer Research Campaign

### Adjuvant therapy for melanoma: how should we respond to high-dose interferon?

**PG de Takats**[1], **MV Williams**[1] **and R Hawkins**[2]

[1]Oncology Centre, Addenbrooke's NHS Trust, Hills Road, Cambridge CB2 2QQ, UK; [2]Division of Oncology, University of Bristol Department of Hospital Medicine, Bristol Oncology Centre, Horfield Road, Bristol BS2 8ED, UK

**Keywords:** melanoma; adjuvant interferon

In 1996, the results were published of a randomized, controlled trial evaluating 'high-dose' interferon alpha 2b (IFN-α) in patients at high risk of recurrence after surgery for thick primary or regional lymph node-positive malignant melanoma (Kirkwood et al, 1996). In this study conducted by the Eastern Cooperative Oncology Group (study E1684), 287 patients were randomized after primary resection to observation alone or to receive IFN-α, 20 MU $m^{-2}$ by 1-h intravenous infusion, days 1–5 for 4 weeks (induction), followed by 10 MU $m^{-2}$ subcutaneously, three times a week for 48 weeks (maintenance).

Analysis of the data at median follow-up of 6.9 years showed a significant prolongation of relapse-free ($P = 0.0023$, one-tailed test) and overall survival ($P = 0.0237$, one-tailed test) in favour of IFN-α therapy. This study is the first and only randomized, prospective trial to date that has shown durable statistically significant survival benefit from any medical therapy after surgery for locally advanced melanoma. In response, the United States Food and Drug Administration granted a licence for adjuvant IFN-α and, since then, the E1684 high-dose IFN-α regimen (alternatively known as the Kirkwood regimen) has become the standard arm in any newly established randomized trial of adjuvant therapy undertaken in the USA in patients with stage IIB and III (American Joint Committee on Cancer staging system, AJCC) malignant melanoma.

IFN-α was licensed for adjuvant use in melanoma in the UK in July 1997. However, significant controversy surrounds the rationale for using this expensive, potentially toxic therapeutic modality. Uncertainty hinges on the requirement for an adequate evidence base justifying treatment. Current clinical status of adjuvant IFN-α – arguments both for and against its use – are outlined here, with the intention of facilitating a move towards a consensus among those involved in melanoma patient care.

### THE PATIENT POPULATION JUSTIFIES INVESTMENT OF RESOURCES

Malignant melanoma currently represents around 1% of all new cancer cases in the UK. Unlike the more common solid tumours, it is a disease often affecting young adults: median age at presentation is below 50 years (OPCS, 1994). Furthermore, the incidence of melanoma is increasing at a rate faster than for any other tumour, currently doubling every 6–10 years. Despite evidence that more individuals are presenting with localized, resectable disease, the death rate is increasing by around 5% per year. Malignant melanoma can behave unpredictably, but studies have shown that, for patients with thick primary tumours (> 4 mm Breslow depth, T4, AJCC stage IIB tumours) and/or regional lymph node involvement (AJCC stage III tumours), over 50% of patients will relapse or die of recurrent disease within 2 years of surgery. When recurrent disease is not amenable to surgery, no medical therapy has yet been shown to impact on survival. Thus, while patients with disseminated disease are considered candidates for phase I trials of novel anti-cancer modalities, there is a clear imperative to define an effective adjuvant therapy that will reduce the risk of disease recurrence after primary melanoma surgery.

*Received 11 August 1997*
*Accepted 14 October 1997*

*Correspondence to:* PG de Takats

### BIOLOGICAL AGENTS HOLD THE GREATEST PROMISE AS SYSTEMIC THERAPY

Conventional cytotoxics tested in patients with advanced disease have been shown to achieve response rates of, at best, 20–25% as single agents and 50–60% in dose-intensive combination regimens (reviewed in McClay and McClay, 1996). However, no chemotherapy regimen to date has been shown to prolong patient survival significantly. Thus, single-agent dacarbazine (response rate around 20%) remains the mainstay of standard therapy outside clinical trials. Early randomized trials of adjuvant therapy in melanoma using dacarbazine (e.g. Kerin et al, 1995), combination chemotherapy (e.g. Meisenberg et al, 1993), bacille Calmette-Guerin (BCG; Tan and Ho, 1993) and other modalities, including levamisole (e.g. Parkinson, 1991; Spitler, 1991) and vitamin A (Meyskens et al, 1994) have not demonstrated any survival advantage with treatment compared with surgery alone.

There is now a wealth of evidence that the immune system can influence the natural history of melanoma, and a variety of biological agents has been shown to possess antitumour activity both in vitro and in vivo. Monoclonal antibodies raised against tumour-specific antigens (e.g. antigens encoded by the MAGE gene family, tyrosinase, Melan-A/MART), active immunotherapy by vaccination with, in particular, melanoma-specific gangliosides, and intensive biochemotherapy regimens are currently being explored in early clinical trials (Balch et al, 1997). Considerable trial experience has already been gained with the type I (IFN-α and -β subtypes) and type II (IFN-γ) interferons as well as interleukin 2 (IL-2), as single agents and in combination regimens, in both the advanced and the adjuvant setting.

**Table 1** Objective response rates observed with interferon alpha in published trials, compared with the most active single-agent chemotherapy drugs

| Agent | Schedule | Evaluable patients | Response (PR + CR) *n* (%) | Reference(s) |
|---|---|---|---|---|
| Dacarbazine | | 1936 | 382 (20) | Balch et al (1993) |
| Fotemustine | | 226 | 56 (25) | McClay and McClay (1996) |
| Cisplatin | | 188 | 43 (23) | Balch et al (1993) |
| Temozolamide | | 56 | 12 (21) | Bleehan et al (1995) |
| IFN-α-2a | 12–50 MU $m^{-2}$ i.m. t.i.w. | 79 | 15 (19) | Creagen et al (1984) Hersey et al (1985) |
| IFN-α-2a | 9–36 MU i.m. Daily | 64 | 4 (11) | Legha et al (1987), Steiner et al (1987), Elsasser-Beile et al (1987) |
| IFN-α-2a | 20 MU $m^{-2}$ i.v. Daily for 5 of 14 days | 15 | 0 (0) | Coates et al (1986) |
| IFN-α-2b | 10 MU s.c. t.i.w. | 22 | 6 (27) | Dorval et al (1986) |
| IFN-α-2b | 10 MU $m^{-2}$ i.m. t.i.w. | 21 | 3 (14) | Sertoli et al (1989) |
| IFN-α-2b | 10 MU $m^{-2}$ s.c. t.i.w. for 12 weeks | 40 | 10 (25) | Robinson et al (1986) |
| IFN-α-2b | 10–100 MU i.m. or i.v. Daily for 28 days | 23 | 5 (22) | Kirkwood et al (1985) |

Recombinant IFN-α has shown broad-spectrum immunomodulatory and antiproliferative activity in a variety of human malignancies, including melanoma. Disappointingly, IFN-α appears to achieve overall response rates no better than the most active single-agent chemotherapy drugs (Tables 1 and 2). However, some 5% of patients consistently achieve complete responses, which appear to be durable, associated with long-term survival (Creagan et al, 1988; Legha, 1997). Despite more than a decade of trial work, the optimal dose and scheduling of IFN-α has not been established. Efficacy and toxicity appear to be both dose and time dependent: higher doses induce more complete responses of longer duration while being more toxic; delayed responses are documented after several months of treatment; a wide variety of recognized IFN-α-related side-effects limit tolerance of treatment (Table 3).

### DOES ADJUVANT INTERFERON OFFER SURVIVAL BENEFIT?

The early trials of adjuvant melanoma immunotherapy failed to show prolongation of patient survival (Fisher et al, 1981; Kaiser et al, 1981; Veronesi et al, 1982), but the role of the recombinant IFNs had yet to be tested. All subtypes of IFN have now been explored to prevent melanoma recurrence after disease resection. IFN-γ has shown minimal anti-tumour activity against melanoma, and the negative randomized trial of adjuvant IFN-γ undertaken by the South West Oncology Group suggested possible adverse effects in the treatment arm (Meyskens et al, 1990, 1995). Several trials of adjuvant IFN-α have been undertaken globally, and Table 4 summarizes completed and ongoing phase III IFN-α studies in high-risk patients. Until now, all such studies have randomized against an observation-only arm.

From current data available from these studies, the value of adjuvant IFN-α remains uncertain. The completed studies of low-dose therapy are not yet mature. However, two such studies have suggested possible patient benefit. The WHO melanoma Program Trial 16 study randomized 444 patients with histologically proven lymph node-positive disease between June 1990 and January 1994 to receive either IFN-α-2a 3 MU flat dose administered subcutaneously (s.c.) three times a week for 3 years, or no treatment after surgery. An early interim analysis indicated highly significant prolongation of disease-free survival for all treated patients and an overall survival benefit for some subgroups (Cascinelli et al, 1994). However, 1 year later, the survival curves converged and the study has not shown durable survival benefit (Cascinelli, 1995). The French Cooperative Group on Melanoma have evaluated the same drug and dose (but treatment period 18 months) in 499 patients with > 1.5-mm-thick primary lesions without clinically detectable lymph node involvement. The first published results, at median follow-up of 2.3 years, indicated no overall survival difference between treated and untreated patients (Grob et al, 1996). However, by June 1995, at median follow-up of 3 years, a statistical prolongation of both relapse-free ($P = 0.029$, two-sided) and overall survival ($P = 0.011$, two-sided) was evident in favour of IFN-α therapy. The results of a 1997 reanalysis are awaited with interest.

The only conventional phase I pharmacokinetic dose-escalation studies with IFN-α were undertaken by ECOG in the early 1980s. Assessing drug administered by either the intravenous (i.v.) or intramuscular (i.m.) route, they determined that 20 MU $m^{-2}$ could be safely given to patients on a daily basis with manageable side-effects (Kirkwood et al, 1985). Thus, trials were undertaken to determine the role of much higher doses of IFN-α in the adjuvant setting.

The results of two high-dose studies have been published to date. E1684 has shown both statistically significant prolongation in disease-free and overall survival with adjuvant IFN-α-2b by 9 months and 12 months respectively (Kirkwood et al, 1996). However, a study by the North Central Cancer Trials Group (NCCTG) randomized 262 patients with primary tumours > 1.69 mm thick or nodal involvement to receive IFN-α-2a 20 MU $m^{-2}$ i.m. three times a week for 12 weeks. At median follow-up of 6.1 years, neither disease-free nor overall survival were significantly greater with treatment (Creagan et al, 1995).

In the UK, currently, standard therapy for stage I–III melanoma remains surgical excision of tumour. Interested, motivated oncologists are likely to be offering high-risk patients entry to one of two ongoing phase III adjuvant IFN-α studies: the UKCCCR-sponsored Aim High study randomizes patients on a 1:1 basis to receive either 'low-dose' IFN-α-2a (3 MU s.c. three times per week for 2 years) or to observation only; the EORTC 18952 study randomizes patients to receive either 'intermediate-dose' IFN-α-2b (10 MU s.c. daily ×5 for 4 weeks, followed by maintenance therapy with either 10 MU s.c. three times per week for 1 year or 5 MU s.c. three times per week for 2 years) vs observation. The EORTC study is skewed such that four in every five patients will receive IFN-α.

Both European studies were initiated at a time when the preliminary results of E1684 were known. At that time, a healthy scepticism among UK oncologists prevailed (Williams et al, 1997); the

© Cancer Research Campaign 1998

**Table 2** Randomized trials comparing interferon alpha plus dacarbazine with dacarbazine in patients with metastatic cutaneous melanoma

| Study | Regimen | No. of patients | Response rate (%) | Median overall survival (months) | |
|---|---|---|---|---|---|
| Falkson et al (1991) | DTIC 200 mg $m^{-2}$ dl–5 vs | 31 | 20 | 9.6 | |
| | DTIC 200 mg $m^{-2}$ dl–5 + IFN-α-2b 15 MU $m^{-2}$ i.v. daily × 3/52, then 10 MU $m^{-2}$ s.c. t.i.w. | 30 | 53 | 17.6 | $P = 0.01$ |
| Thomson et al (1993) | DTIC 800 mg $m^{-2}$ dl vs | 83 | 17 | 7.5 | |
| | DTIC 200–800 mg $m^{-2}$ dl + IFN-α-2a 3–9 MU $m^{-2}$ s.c. t.i.w. | 87 | 21 | 8.8 | NS |
| Bajetta et al (1994) | DTIC 800 mg $m^{-2}$ dl q3/52 vs | 82 | 20 | 11 | |
| | DTIC 800 mg $m^{-2}$ dl q3/52 + IFN-α-2a 3 MU i.m. t.i.w. vs | 84 | 23 | 11 | |
| | DTIC 800 mg $m^{-2}$ dl q3/52 + IFN-α-2a 9 MU i.m. daily | 76 | 28 | 13 | NS |
| Falkson et al (1996) | DTIC 200 mg $m^{-2}$ dl–5 vs | 67 | 12 | | |
| | DTIC 200 mg $m^{-2}$ dl–5 + IFN-α-2b 15 MU $m^{-2}$ i.v. daily for 3/52, then 10 MU $m^{-2}$ s.c. t.i.w. | 65 | 21 | – | NS |

DTIC, dacarbazine; t.i.w., three times a week; NS, no statistically significant difference.

**Table 3** Recognized side-effects associated with interferon alpha therapy, in order of frequency

Flu-like symptoms
Fatigue/lethargy
Neutropenia
Lymphopenia
Fever
Myalgia
Anorexia
Nausea/vomiting
Elevated liver transaminases
Headache
Chills/rigors
Depression
Altered mood
Loss of concentration
Diarrhoea
Abdominal pain
Alopecia
Altered taste sensation
Dizziness/vertigo
Anaemia

Rarely
- Jaundice
- Liver failure
- Severe psychological disturbance (suicidal depression)
- Behavioural changes
- Confusion
- Cortical blindness
- Retinopathy
- Leukoencephalopathy

ECOG study randomized a small number of patients ($n$ = 287) and analysed the data using a one-tailed Student's $t$-test, so presupposing that treatment would be superior to no treatment. The toxicity associated with IFN-α doses used by ECOG was excessive by European standards: more than two out of three patients overall required dose reduction or delay and two patients died 1 and 3 months into treatment from liver failure. Notably, when the original study data were reanalysed taking into account toxicity, overall quality of life adjusted survival was no longer statistically significant using a two-tailed test (Cole et al, 1996).

Recruitment to these European studies has been disappointingly poor. By October 1997, 2 years after its launch, 212 patients had been entered from 23 UK centres in to Aim High: a study that requires 1000 patients to define a 10% difference in recurrence-free and overall survival (90% power). Recruitment to the EORTC study is more promising, averaging 40–50 patients per month, and is predicted to achieve the recruitment target of 1000 patients by the year 1999. However, few centres from the UK are actively contributing to this study. So what is happening to eligible patients? It appears that the Kirkwood regimen is being increasingly prescribed outside of any clinical trial, both as a consequence of pressure from well-informed patients surfing the internet, but also because oncologists have been convinced by the results of a single randomized trial.

### IS ONE STUDY ENOUGH?

Any new treatment should be established by comparison against current standard therapy in a randomized prospective controlled trial. However, as indicated by E1684, the design of any one study may itself be considered to be flawed and, therefore, convention requires that two independent studies are required to justify change of practice. Even then, a statistical $P$-value cannot in itself define the new standard – for example, exactly how much longer do patients live with treatment? What are the costs to patients in terms of quality of life (Cole et al, 1996)? What are the resource implications of introducing the new therapy (Hillner et al, 1997)? – we must account for cost of drug, plus drug-induced toxicity that may include prophylactic medication, hospital admissions and days lost from work. And to what extent must we consider the opportunity cost for other patients in a resource-limited health system? Appropriately, after closing the first study, ECOG undertook a

© Cancer Research Campaign 1998

**Table 4** Completed and current randomized trials of adjuvant interferon alpha in patients with resected malignant melanoma at high risk of recurrence

| Trial | Eligible patients[a] | Interferon schedule | Planned total dose | Total no. of patients | Comments |
|---|---|---|---|---|---|
| ECOG EST 1684 | T4, N1 | IFN-α-2b induction: 20 MU $m^{-2}$ daily i.v. × 4 weeks then maintenance: 10 MU $m^{-2}$ t.i.w. s.c. × 48 weeks | 3500 MU | 287 | Significant difference in DFS: 1.72 vs 0.9 years ($P = 0.004$) Significant difference in OS: 3.82 vs 2.78 years ($P = 0.046$) |
| Intergroup study[b] | T4, N1 | IFN-α-2b induction: 20 MU $m^{-2}$ daily i.v. × 4 weeks, then maintenance: 10 MU $m^{-2}$ t.i.w. s.c. × 48 weeks<br>vs 3 MU t.i.w. s.c. × 2 years | 3500 MU<br>1400 MU | 642 | Study closed – results awaited May 1998 |
| NCCTG 83-7052 | T3–4, N1 | IFN-α-2b 20 MU $m^{-2}$ daily i.m. × 3 months | 2210 MU | 262 | No significant difference in DFS: 2.7 vs 2 years ($P = 0.19$) or OS: 6.6 vs 5 years ($P = 0.40$) |
| EORTC 18952 | T4, N1 | IFN-α-2b induction: 10 MU daily s.c. × 4 weeks then maintenance: 10 MU t.i.w. s.c. × 1 year or 5 MU t.i.w. s.c. × 2 years | 1760 MU | 1000[c] | Continues to recruit in Europe |
| WHO 16 | T4, N1–2 | IFN-α-2a 3 MU t.i.w. s.c. × 3 years | 1400 MU | 444 | At median follow-up 3.25 years no significant difference in DFS or OS |
| Aim High | T4, N1–2 | IFN-α-2a 3 MU t.i.w. s.c. × 2 years | 936 MU | 1000[c] | Continues to recruit in UK |
| French Consortium | T3–4 | IFN-α-2a 3 MU t.i.w. s.c. × 18 months | 702 MU | 499 | At median follow-up 3 years, significant difference in DFS ($P = 0.029$) and OS ($P = 0.011$) |
| Austrian Melanoma Cooperative | T3–4 | IFN-α-2a induction: 3 MU daily s.c. × 3 weeks then maintenance: 3 MU t.i.w. s.c. × 1 year | 513 MU | | No interim analysis yet |
| Scottish Melanoma Group | T3–4 | IFN-α-2a 3 MU t.i.w. s.c. × 6 months | 234 MU | 92 | No survival difference to date |
| EORTC 18871 | T3–4, N1–2 | IFN-α-2b 1 MU alt days s.c. × 1 year vs IFN-γ × 1 year | 182 MU | 830 | Closed April 1996: no survival difference so far |

[a]American Joint Committee on Cancer staging system (1992): T3 is stage IIA, T4 is stage IIB, N1,2 is stage III. [b]Intergroup study: ECOG E1690/SWOG9111/CALGB9190. N.B. this study differs from E1684 in that lymphadenectomy was required for patients with T4 primary melanomas in E1684, but not required in the later trial. [c]Planned total recruitment number. References for published studies: Kirkwood et al (1996) (ECOG 1684); Creagen et al (1995) (NCCTG 83-7052); Cascinelli (1995) (WHO 16).

second adjuvant melanoma trial as part of an Intergroup trial (E1690/SWOG9111/CALGB9190), which randomized 642 patients after melanoma resection to receive either the Kirkwood regimen, low-dose IFN-α as per the Aim High study or to observation. The results of this pivotal study will become known later this year and, clearly, will determine whether high-dose IFN-α is the treatment of choice for these patients.

### DETERMINING STANDARD ADJUVANT THERAPY

While awaiting the results of E1690, given the time and effort required to organize a multicentre trial, it is necessary to plan well in advance. We can do this by considering our response to the possible outcomes of this study. Let us suppose that E1690 shows no statistical survival benefit of high-dose IFN-α over either low-dose IFN-α or observation. Both purchasers and providers could be forgiven for breathing a heavy sigh of relief – doctors and their patients will be disappointed.

Is there a chance that the low-dose IFN-α arm could show benefit over observation? Given the current analysis of the French Consortium study, this possibility should not be ruled out. It is probably reasonable to assume that, as long as the high-dose IFN-α arm of E1690 does not show statistical survival benefit over either other arm, UK oncologists would be justified in continuing to support both Aim High and EORTC 18952, in which both studies address other key end points, such as quality of life, health economics and molecular prognostic indicators in addition to patient survival. Our ability to contribute to such important clinical trials, however, could potentially be seriously impeded as aconsequence of premature drug

© Cancer Research Campaign 1998

licensing. Cancer centres will find it difficult to absorb the cost of IFN-α, estimated to be approximately £10 000 and £5500 per treated patient within the EORTC 18952 and Aim High studies respectively. The predicament of resourcing clinical research involving expensive drugs must be raised both with the public and the politicians as a matter of urgency if we are to fulfil our ethical and medical obligations to patients.

Now let us suppose that E1690 clearly shows survival benefit with high-dose IFN-α over low-dose therapy and observation. The criteria for defining a new standard therapy will be met and oncologists will be duty-bound to address key issues: is it unethical not to offer some form of IFN-α to patients at high risk and, by the same argument, therefore to continue to recruit to the European adjuvant studies? If ethically duty-bound to treat, do we realistically have access to the necessary resources – finances, specialist staff, in-patient and out-patient facilities? a pharmacoeconomic analysis undertaken on the basis of E1684 (Hillner et al, 1997) has calculated the cost of high-dose IFN-α per life year gained to be around $13 700 after 35 years. This figure equates with the cost-effectiveness of adjuvant chemotherapy for node-negative breast cancer, while being 30–50% cheaper than taxol used as first-line therapy in ovarian cancer. However, the absolute drug cost of around £20 000 per treated patient will not be easily forthcoming from purchasers within the UK.

### THE WAY FORWARD

With the knowledge available to us, one could apply forethought and consider future initiatives in management of high-risk melanoma patients. Are we yet ready to design the next adjuvant study should E1690 confirm survival benefit with high-dose IFN-α? Alternatively, has the patient population who may benefit from adjuvant therapy been adequately defined?

One obvious key question relates to the scientific rationale for high-dose IFN-α as defined by the Kirkood regimen. The mechanism by which high-dose therapy actually works is unknown. To our knowledge, no true dose–response relationship has been demonstrated for IFN-α in terms of immune modulation. It has been argued that the route of administration of the induction phase in the Kirkwood regimen – i.v. rather than s.c. – is the key to efficacy. The original pharmacokinetic study (Kirkwood et al, 1985) showed that, while IFN-α was detectable in serum after 24 h only after i.m. administration, significantly higher peak plasma concentrations were achieved with i.v. drug administration. As the survival curves separated early in E1684, the high peak plasma levels achieved by i.v. drug administration in the first month may confer the pharmacological advantage in eradicating micrometastatic disease. This theory has, however, yet to be adequately substantiated. Furthermore, although the randomized NCCTG trial of short-term, high-dose i.m. IFN-α (Creagan et al, 1995) is usually regarded as showing no benefit over surgery alone, the difference in median disease-free survival for node-positive patients (17 vs 10.8 months) was significant when adjusted for other parameters in a Cox model ($P = 0.04$). Taken together, there appears good justification for evaluating the benefits of short-term, high-dose IFN-α regimens compared with the 1-year Kirkwood regimen. If equivalence could be proven, then savings in terms of drug costs, hospital resources, patient toxicity and quality of life are likely to be substantial. Such a result might be pertinent to the management of other tumour types, such as lymphomas and renal cell carcinomas, in which IFN-α plays a role.

It might be possible to build into such an equivalence study an assessment of survival benefit by incorporating other strategies of immune modulation. For example, serology studies have shown that patients who develop antibodies against melanoma-specific cell-surface glycolipids, known as gangliosides, have a favourable prognosis. The purified ganglioside GM2 has been administered to patients with an immune adjuvant (BCG) to augment the antibody response elicited by GM2 antigen. In a randomized trial conducted in 122 stage III melanoma patients (Livingstone et al, 1994), induction of anti-GM2 IgM antibodies was demonstrated in a majority of treated patients. Vaccination was associated with negligible patient toxicity and borderline prolongation of relapse-free survival. Since this early study, more efficient carrier systems and potent immune adjuvants have been developed. Thus, GM2–KLH/QS-21 vaccine (GM2 conjugated to keyhole limpet haemocyanin and administered with the immune adjuvant QS-21) is currently being compared against the Kirkwood regimen in the USA Intergroup trial of adjuvant therapy in stage IIB and III malignant melanoma patients. The same ganglioside vaccine is about to be tested against observation in patients with resected stage IIA (1.5–4 mm thick) melanomas in Europe. Such vaccines, being cancer specific and evidently non-toxic to-patients, afford potential for coupling with IFN-α, but adjuvant combination studies have as yet not been undertaken.

Finally, it is clear that only a small subset of patients who received high-dose IFN-α in E1684 derived benefit from it. Identification of surrogate markers of response will be important as both future prognostic and therapeutic tools. In E1684, all patients underwent elective lymph node dissection, which provided useful staging information, but its role in terms of survival benefit is controversial. The technique of lymphatic mapping and sentinel node biopsy was introduced by Morton et al (1992) as a means of detecting occult nodal metastases, and thus the offer of selective lymphadenectomy to those patients. Now combined with intraoperative use of a gamma probe to detect injected radioactive colloid in addition to blue dye, experienced operators are able to identify the sentinel node in 99% of cases. Various investigators have developed reverse transcription polymerase chain reaction methods to sensitively and specifically measure tyrosinase mRNA in sentinel lymph nodes (Wang et al, 1994) and to detect circulating melanoma cells in blood (Smith et al, 1991; Brossart et al, 1995). The current USA and EORTC adjuvant studies are undertaking measurement of such molecular markers in primary tumour, lymph node tissue and blood of trial patients. What impact these kind of techniques will have in terms of patient selection for therapeutic intervention and subsequent survival outcome remains to be determined.

### FINAL COMMENT

There is accumulating evidence demonstrating that the host's immunity influences melanoma behaviour. Recent clinical demonstration of survival benefit with adjuvant high-dose IFN-α, albeit in a single randomized clinical trial, was sufficient to justify licensing of IFN-α for adjuvant use in the UK and to persuade a growing number of specialists that this is the treatment of choice for selected patients at high risk of disease recurrence and, ipso facto, virtual certain death. But before oncologists protest the imperative for allocating limited resources to fund IFN-α, we must be sure that the quality of evidence in favour of treatment justifies the cost both to the health service and to the patient. If, on

© Cancer Research Campaign 1998

reviewing the evidence, uncertainty prevails, we have a duty to address the issues surrounding adjuvant melanoma therapy in a systematic, controlled manner within the context of well-designed clinical trials. Until such time as E1690 has been analysed, ongoing adjuvant studies (Aim High and EORTC 18952) should be supported, and the argument for financing of IFN-α justified in terms of facilitating clinical research of fundamental importance.

Experience with adjuvant melanoma trials performed to date suggests that patient recruitment is difficult. Thus, for future trials, co-operation with our USA and European colleagues is essential. By gaining unanimous support from within the oncology disciplines, we may better influence government, funding agencies, purchasers and industry for the right drug to be available to the right people at the right time.

### REFERENCES

Bajetta E, Di Leo A, Zampino MG, Sertoli MR, Comella G, Barduagni M, Giannotti B, Queirolo P, Tribbia G, Bernengo MG, Menichetti ET, Palmeri S, Russo A, Cristofolini M, Erbazzi A, Fowst C, Criscuolo D, Bufalino R, Zilembo N and Cascinelli N (1994) Multicentre randomized trial of dacarbazine alone or in combination with two different schedules of interferon alfa-2a in the treatment of advanced melanoma. *J Clin Oncol* **12**: 806–811

Balch CM, Houghton AN and Peters LJ (1993) Cutaneous melanoma. In *Cancer: Principles and Practice of Oncology*, De Vita VT, Hellman S and Rosenberg SA. (eds), pp. 1612–1661. JB Lippincott: Philadelphia

Balch CM, Reintgen DS, Kirkwood JM, Houghton A, Peter L and Ang KK (1997) Cutaneous melanoma. In *Cancer: Principles and Practice of Oncology*, DeVita VT, Hellman S and Rosenberg SA. (eds) pp. 1947–1994. Lippincott–Raven Publishers: Philadelphia

Bleehan NM, Newlands ES, Lee SM, Thatcher N, Selby P, Calvert AH, Rustin GJS, Brampton M and Stevens MFG (1995) Cancer Research Campaign phase II trial of temozolamide in metastatic melanoma. *J Clin Oncol* **13**: 910–913

Brossart P, Schmier J, Kruger S, Willhauck M, Scheibenbogen C, Mohler T and Keilholz U (1995) A polymerase chain reaction-based semiquantitative assessment of malignant melanoma cells in peripheral blood. *Cancer Res* **55**: 4065–4068

Cascinelli N (1995) Evaluation of efficacy of adjuvant rIFNα 2a in melanoma patients with regional node metastases (abstract). *Proc Am Soc Clin Oncol* **14**: 410

Cascinelli N, Bufalino R, Morabito A and Mackie R (1994) Results of adjuvant interferon study in WHO melanoma programme (letter). *Lancet* **343**: 913–914

Coates A, Rallings M, Hersey P and Swanson C (1986) Phase II study of recombinant alpha 2-interferon in advanced malignant melanoma. *J Interferon Res* **6**: 1–4

Cole BF, Gelber RD. Kirkwood JM, Goldhirsch A, Barylak E and Borden E (1996) Quality-of-life-adjusted survival analysis of interferon alfa-2b adjuvant treatment of high-risk resected cutaneous melanoma: an Eastern Cooperative Oncology Group study. *J Clin Oncol* **14**: 2666–2673

Creagan ET, Ahmann DL, Green SJ, Long HJ, Rubin J, Schutt AJ and Dziewanowski ZE (1984) Phase II study of low-dose recombinant leukocyte A interferon (rIFN-alpha A) in disseminated malignant melanoma. *Cancer* **54**: 2844–2849

Creagan ET, Loprinzi CL, Ahmann DL and Schaid DJ (1988) A phase I–II trial of the combination of recombinant leukocyte A interferon and recombinant human interferon-gamma in patients with metastatic malignant melanoma. *Cancer* **62**: 2472–2474

Creagan ET, Dalton RJ, Ahmann DL, Jung S-H, Morton RF, Langdon RM, Kugler J and Rodrigue LJ (1995) Randomized, surgical adjuvant trial of recombinant interferon-alfa-2a in selected patients with malignant melanoma. *J Clin Oncol* **13**: 2776–2783

Dorval T, Palangie T, Jouve M, Garcia-Giralt E, Israel L, Falcoff E, Schwab D and Pouillart P (1986) Clinical phase II trial of recombinant DNA interferon (interferon alfa 2b) in patients with metastatic malignant melanoma. *Cancer* **58**: 215–218

Elsasser-Beile U and Drews H (1987) Interferon in the treatment of malignant melanoma: results of clinical studies. *Fortschr Med* **105**: 401–403

Falkson CI, Falkson G and Falkson H (1991) Improved results with the addition of interferon alfa-2b to dacarbazine in the treatment of patients with metastatic malignant melanoma. *J Clin Oncol* **9**: 1403–1408

Falkson CI, Ibrahim J, Kirkwood J and Blum R (1996) A randomized phase III trial of dacarbazine (DTIC) versus DTIC + interferon alfa2b (rIFN) versus DTIC + tamoxifen (TMX) versus DTIC + rIFN + TMX in metastatic malignant melanoma: and ECOG trial (abstract). *Proc Am Soc Clin Oncol* **15**: 435

Fisher RI, Terry WD, Hodes RJ, Rosenberg SA, Makuch R, Gordon HG and Fisher SG (1981) Adjuvant immunotherapy or chemotherapy for malignant melanoma: preliminary report of the National Cancer Institute randomized clinical trial. *Surg Clin North Am* **61**: 1267–1277

Grob JJ, Dreno B, Delaunay M, Chastang C, Guillot B, Cupissol B, Souteyrand P, Sassolas B, Cesarini JP, Thivolet J, Denoeux JP, Ortanne JP, Thomas L, Beylot C, Truchetet F, Lovette G, Chemaly C, Meynadier J, Amblard P, Thyss P, Avril MF, Prigent F and Bonerandi JJ (1996) Results of the French multicentre trial on adjuvant therapy with interferon alfa-2a in resected primary melanoma (> 1.5 mm) (abstract). *Proc Am Soc Clin Oncol* **15**: 437

Hersey P, Hasic E, Macdonald M, Edwards A, Spurling A, Coates AS, Milton GW and McCarthy WH (1985) Effects of recombinant leukocyte interferon (rIFN-alpha A) on tumour growth and immune responses in patients with metastatic melanoma. *Br J Cancer* **51**: 815–826

Hillner BE, Kirkwood JM, Atkins MB, Johnson ER and Smith TJ (1997) Economic analysis of adjuvant interferon-alfa 2b in high risk melanoma based on projections from Eastern Cooperative Oncology Group 1684. *J Clin Oncol* **15**: 2351–2358

Kaiser LR, Burk MW and Morton DI (1981) Adjuvant therapy for malignant melanoma. *Surg Clin North Am* **61**: 1249–1257

Kerin MJ, Gillen P, Monson JR, Wilkie J, Keane FB and Tanner WA (1995) Results of a prospective randomized trial using DTIC and interferon as adjuvant therapy for stage I malignant melanoma. *Eur J Surg Oncol* **21**: 548–550

Kirkwood JM, Ernstoff MS, Davis CA, Reiss M, Ferraresi R and Rudnick SA (1985) Comparison of intramuscular and intravenous recombinant alpha-2 interferon in melanoma and other cancers. *Ann Intern Med* **103**: 32–36

Kirkwood JM, Strawderman MH, Ernstoff MS, Smith TJ, Borden EC and Blum RH (1996) Interferon alfa-2b adjuvant therapy of high-risk resected cutaneous melanoma: the Eastern Cooperative Oncology Group Trial EST 1684. *J Clin Oncol* **14**: 7–17

Legha SS (1997) The role of interferon alfa in the treatment of metastatic melanoma. *Sem Oncol* **24**: 24–31

Legha SS, Papadopoulos NE, Plager C, Ring S, Chawla SP, Evans LM and Benjamin RS (1987) Clinical evaluation of recombinant interferon alfa-2a (Roferon-A) in metastatic melanoma using two different schedules. *J Clin Oncol* **5**: 1240–1246

Livingstone PO, Wong GYC, Adluri S, Tao Y, Padavan M, Parente R, Hanlon C, Calves MJ, Helling F, Ritter G, Oettgen HF and Old LJ (1994) Improved survival in stage III melanoma patients with GM2 antibodies: a randomized trial of adjuvant vaccination with GM2 ganglioside. *J Clin Oncol* **12**: 1036–1044

McClay EF and McClay M-ET (1996) Systemic chemotherapy for the treatment of metastatic melanoma. *Sem Oncol* **23**: 744–753

Meisenberg BR, Ross M, Vredenburgh JJ, Jones R, Shpall EJ, Seigler HF, Coniglio DM, Wu K and Peters WP (1993) Randomized trial of high-dose chemotherapy with autologous bone marrow support as adjuvant therapy for high-risk, multi-node positive malignant melanoma. *J Natl Cancer Inst* **85**: 1080–1085

Meyskens FL Jr, Kopecky K, Samson M, Hersh E, Macdonald J, Jaffe H, Crowley J and Coltman C (1990) Recombinant human interferon-γ: adverse effects in high-risk stage I and II cutaneous malignant melanoma (letter). *J Natl Cancer Inst* **82**: 1071

Meyskens FL, Liu P, Tuthill RJ, Sondak VK, Fletcher WS and Jewell WR (1994) Randomized trial of vitamin A versus observation as adjuvant therapy in high-risk primary malignant melanoma: a Southwest Oncology Group study. *J Clin Oncol* **12**: 2060–2065

Meyskens FL Jr, Kopecky KJ, Taylor CW, Noyes RD, Tuthill RJ, Hersh EM, Feun IG, Doroshow JH, Flaherty LE and Sondak VK (1995) Randomized trial of adjuvant human interferon gamma versus observation in high-risk cutaneous melanoma: a Southwest Oncology Group study. *J Natl Cancer Inst* **87**: 1710–1713

Morton DL, Wen DR, Wong JH, Economou JS, Cagle LA, Storm FK, Foshag LJ and Cochran AJ (1992) Technical details of intraoperative lympathic mapping for early-stage melanoma. *Arch Surg* **127**: 392–399

Office of Population Censuses and Surveys (1994) *Cancer statistics: registrations of cancer diagnosed in 1988, England and Wales.* HMSO: London

Parkinson DR (1991) Levamisole as adjuvant therapy for melanoma: quo vadis? *J Clin Oncol* **9**: 2052

Robinson WA, Mughal TI, Thomas MR, Johnson M and Spiegel RJ (1986) Treatment of metastatic malignant melanoma with recombinant interferon alpha 2. *Immunobiology* **172**: 275–282

© Cancer Research Campaign 1998

Sertoli MR, Bernengo MG, Ardizzoni A, Brunetti I, Falcone A, Vidili MG, Cusimano MP, Appino A, Doveil G and Fortini C (1989) Phase II trial of recombinant alpha-2b interferon in the treatment of metastatic skin melanoma. *Oncology* **46**: 96–98

Smith B, Selby P, Southgate J, Pitman K, Bradley C and Blair GE (1991) Detection of melanoma cells in peripheral blood by means of reverse transcriptase and polymerase chain reaction. *Lancet* **388**: 1227–1229

Spitler LE (1991) A randomized trial of levamisole versus placebo as adjuvant therapy in malignant melanoma. *J Clin Oncol* **9**: 736–740

Steiner A, Wolf C and Pehamberger H (1987) Comparison of the effects of three different treatment regimens of recombinant interferons (rIFN alpha, rIFN gamma and rIFN alpha + cimetidine) in disseminated malignant melanoma. *J Cancer Res Clin Oncol* **113**: 459–465

Tan JK and Ho VC (1993) Pooled analysis of the efficacy of bacille Calmette-Guerin (BCG) immunotherapy in malignant melanoma. *J Dermatol Surg Oncol* **19**: 985–990

Thomson DB, Adena M, McCleod GR, Hersey P, Gill PG, Coates AS, Olver In, Kefford RF and Lowenthal RM (1993) Interferon alpha 2a does not improve response or survival when combined with dacarbazine in metastatic malignant melanoma: results of a multi-institutional Australian randomized trial. *Melanoma Res* **3**: 133–138

Veronesi U, Adamus J, Aubert C, Bajetta E, Beretta G, Bonadonna G, Bufalino R, Cascinelli N, Cocconi G, Durand J, De Marsillac J, Ikonopisov RL, Kiss B, Lejeune F, Mackie R, Madej G, Mulder H, Mechl Z, Milton GW, Morabito A, Peter H, Priario J, Paul E, Rumke P, Sertoli R and Tomin R (1982) A randomised trial of adjuvant chemotherapy and immunotherapy in cutaneous melanoma. *New Engl J Med* **307**: 913–916

Wang X, Heller R, Vanvoorhis N, Cruse CW, Glass F, Fenske N, Berman C, Leo-Messina J, Rappaport D and Wells K (1994) Detection of submicroscopic metastases with polymerase chain reaction in patients with malignant melanoma. *Ann Surg* **220**: 768–774

Williams MV, de Takats PG and Parmer M (1997) Survival benefit in melanoma. *J Clin Oncol* **15**: 2172

© Cancer Research Campaign 1998